# Bibliography

**Published:** 1900-12-15

**Authors:** 


### BIBLIOGRAPHY.

The correction of irregularities of the teeth or the practice of Orthodontia has to some extent become a specialty of dental practice, and a few men are giving their entire time to its practice, and with the most satisfactory results. Perhaps no one in this or any other country has done more to develop the specialty of Orthodontia than Dr. E. H. Angle. We are pleased to announce that he has just issued the sixth edition of his work on this subject, under the title of "The Treatment of Malocclusion of the Teeth and Fractures of the Maxillæ." While the work is devoted to an exposition almost entirely of the author's methods, he has embodied in it a better classification of methods and principles for practical procedures than can be found in any other existing treatise. His definition, for instance, of Orthodontia is unique and happy; he calls it the science which has for its object the correction of malocclusion of the teeth. He attributes all irregularities to malocclusion and states a sensible formula for a correct occlusion and a nomenclature to more clearly express the various deviations from the truly normal which is worth studying not only by those who wish to specialize in this subject, but it might very well be utilized in Prosthetic Dental Art as well.

The author suggests the inherited malpositions of the teeth are not only possible, but are frequently intensified. If true, this is a very important matter and increases materially the responsibility we as dentists hold toward our patients, in regard to their present happiness and the welfare of future generations.

The chapter on diagnosis is one of the most valuable in the book. If the principles stated and suggestions made were more fully worked out and mastered by every one attempting this work, fewer mistakes in designing appliances and less failures would be made, resulting from a proper appreciation at the start of actual conditions and the possibilities of attaining ideal conditions by a proper interference. The author suggests that the day of extempore regulating appliances has gone by, and that with the present methods it is practicable to correct all forms of malocclusion with appliances which can be had of the dealers in supplies ready or with very slight alterations to be applied to the case in hand. To this statement we are not quite ready to assent, but in the main it may be true. We have no doubt many failures to accomplish desirable results are due not so much to the methods employed as to apparatus improperly designed or carelessly made. The classification of cases for treatment is a step in the right direction, but we are inclined to think that the author has so simplified his classification as to make it somewhat incomprehensible to one who has not taken time to try to bring the almost endless varieties of cases met under its orders. Dr. Angle's wide experience ought, however, to entitle him to a higher order of judgment in this matter than our hasty consideration deserves.

There is much that is deserving in the work that we would like to commend, but we find our space will not permit of further comment. We believe it is one of the best works on this subject that has yet appeared, and it should be in the library of every one who makes any attempt to do this class of work.

It is beautifully printed and illustrated with a large number of entirely new cuts detailing the author's methods with cases by classes and the technique of appliances. It is published by the S. S. White Dental Manufacturing Company, and does this house great credit. It is an octavo volume of 300 pages and costs $4.00.

---

THE sixth edition of "Evan's Crown and Bridge-work" has also just come to hand from the publishing department of the S. S. White Dental Manufacturing Company. This standard work is so well known that it hardly needs any commendation from us. The development of this subject has been so rapid that frequent editions of this work have been necessary to keep the book abreast of the actual condition of the subject. But fortunately the work of Dr. Evans is so popular that the publishers have no excuse for not issuing new editions at so frequent intervals as to keep the book well abreast of the times.

The book is about the same in size as the former edition, although quite a little new matter has been added. The chapter on porcelain work is a new feature. We do not think it is given as much space as its importance deserves, but the chapter contains a brief sketch at least of the more popular methods. We trust that by the time the next edition is needed this subject shall have been passed from its present somewhat indefinite practice to that of uniform and systematic methods. A large part of the space devoted to the methods of constructing extensive bridges with anchorages to a few teeth might be given over to more important details. If experience has taught us anything in bridge construction, it is that extensive bridge-work with few anchorages are bound to fail too soon to justify the necessarily expensive construction.

As a treatise on this subject, the work has many shortcomings. It will serve a good purpose, however, as a text-book for class work and a reference book for special methods for practitioners.

